# Power flow analysis and optimal locations of resistive type superconducting fault current limiters

**DOI:** 10.1186/s40064-016-3649-4

**Published:** 2016-11-14

**Authors:** Xiuchang Zhang, Harold S. Ruiz, Jianzhao Geng, Boyang Shen, Lin Fu, Heng Zhang, Tim A. Coombs

**Affiliations:** 1 Department of Engineering, University of Cambridge, 9 JJ Thomson Avenue, Cambridge, CB3 0FA UK; 2 Department of Engineering, University of Leicester, University Road, Leicester, LE1 7RH UK

**Keywords:** Superconducting fault current limiter, Distributed power system, Short-circuit current, Optimal location

## Abstract

Based on conventional approaches for the integration of resistive-type superconducting fault current limiters (SFCLs) on electric distribution networks, SFCL models largely rely on the insertion of a step or exponential resistance that is determined by a predefined quenching time. In this paper, we expand the scope of the aforementioned models by considering the actual behaviour of an SFCL in terms of the temperature dynamic power-law dependence between the electrical field and the current density, characteristic of high temperature superconductors. Our results are compared to the step-resistance models for the sake of discussion and clarity of the conclusions. Both SFCL models were integrated into a power system model built based on the UK power standard, to study the impact of these protection strategies on the performance of the overall electricity network. As a representative renewable energy source, a 90 MVA wind farm was considered for the simulations. Three fault conditions were simulated, and the figures for the fault current reduction predicted by both fault current limiting models have been compared in terms of multiple current measuring points and allocation strategies. Consequently, we have shown that the incorporation of the *E*–*J* characteristics and thermal properties of the superconductor at the simulation level of electric power systems, is crucial for estimations of reliability and determining the optimal locations of resistive type SFCLs in distributed power networks. Our results may help decision making by distribution network operators regarding investment and promotion of SFCL technologies, as it is possible to determine the maximum number of SFCLs necessary to protect against different fault conditions at multiple locations.

## Background

With the persistent increase of conventional system generation and distributed generations (DGs), such as photovoltaic plants, concentrating solar power plants, and wind farms, the likelihood of fault events capable of causing great and irreparable damage to a large set of electrical devices, or even system blackouts, has been rapidly rising (Zhang et al. 2013; Zheng et al. 2015). Various strategies for mitigating fault current levels have been implemented in the power industry, such as construction of new substations, splitting existing substation buses, upgrading of multiple circuit breakers, and the installation of high impedance transformers. Nevertheless, all these operational practices involve a non-negligible degradation of the systems stability and performance, which ultimately means the occurrence of significant economic losses and further investment (Kovalsky et al. 2005). Series reactors and solid state fault current limiters are also widely used, although these insert a high impedance causing a continuous voltage drop and power losses during normal operation (Ye and Juengst 2004). However, superconducting fault current limiting technology can stand up to all these difficulties, preserving the stability and reliability of the power system with minimum losses under normal conditions (Angeli et al. 2016), although a comparison of different fault protection approaches is out of the scope of this manuscript. An exhaustive review on successful field tests and different existing numerical models of SFCLs can be found in Ref. Ruiz et al. (2015).

Two simplified SFCL models have been identified as in common use for simulating the performance of SFCLs installed in real power grids. The first approach is to model the SFCL as a step-resistance with a pre-defined triggering current, quench time, and recovery time, as in Ref. Khan et al. (2011) and Ref. Hwang et al. (2013). This approach allows us to consider a simplified scenario where no energy loss occurs during the superconducting state and a high impedance in normal state, by assuming that the SFCL responds to faults instantaneously. However, this may lead to significant inaccuracies, since the quenching and recovery characteristics depend on the thermal and electrical properties of the superconductors, which are both neglected in this simplified model. Modelling of a resistive type SFCL can also be simplified by using an exponential function for the dynamic resistance of the SFCL device, in which the quenching action of the superconducting material is solely determined by time. This method has been previously implemented in Ref. Park et al. (2010, 2011) in order to study the optimal locations and associated resistive values of SFCLs for a schematic power grid with an interconnected wind-turbine generation system, which found that the installation of SFCLs cannot only reduce the short-circuit current level, but also dramatically enhance the reliability of the wind farm. Compared to the previous approach, this exponential resistance curve fits better with the real performance of an SFCL and furthermore provides aggregated computational benefits in terms of numerical convergence. Nevertheless, SFCL characteristics, including triggering current, quenching, and recovery time, must also be set before initialising the simulation. Therefore, under this scenario the physical properties of the superconductors are ignored as well. A more advanced model for a resistive-type SFCL was presented in Ref. Langston et al. (2005), in which both the physical properties and the real dimensions of superconductors were considered. A similar model was then built by Colangelo and Dutoit (2013) in order to simulate the behaviour of the SFCL designed in the ECCOFLOW project. Using this model the quenching action of the SFCL is no longer pre-defined. However, the computational complexity of these models is significantly increased, especially during large scale power network simulations. Hence, during a performance simulation of SFCLs installed in power systems, it is important to study the necessity of considering the thermal and electrical properties of superconducting materials, in order to be able to choose a better trade-off between computational complexity and model accuracy. For any of the adopted strategies, the research must ultimately address the process of finding of the optimal locations for multiple SFCLs inside a power network, which, according to our knowledge, has yet only considered a maximum of just two SFCLs. This means that the cooperation between prospective need for more SFCLs remains an open issue.

In this paper we present a comprehensive study into the performance and optimal location analysis of resistive type SFCLs in realistic power systems, starting from the simplest consideration of a single step-resistance for the activation of an SFCL, up to considering the actual electro-thermal behaviour of the superconducting component. We have simulated the performance of SFCLs described by two different models: (1) as a non-linear resistance depending on time, and (2) as a dynamic temperature-dependent model consisting of the actual *E*–*J* characteristics of the superconducting material. The applied power grid model which includes interconnected dispersed energy resources was built based on the UK network standard. Through simulation of the system behaviours under three fault conditions (two distribution network faults in different branches, and one transmission system fault), the optimum SFCL installation schemes were found from all the feasible combinations of SFCLs. In addition, a detailed comparison between the figures obtained for each of the above cases was performed, proving that the non-linear resistor model is insufficient for accurate estimation of the reliability and optimal location of an SFCL, as the complex thermal and electrical behaviours of the superconducting material during its transition to the normal state cannot be simplified to a single step-resistance.

This paper is organised as follows. “SFCLs and topology of the power system” section introduces the topology of the power system and the proposed SFCL models. “Network stability, current limiting performance, and recovery characteristics” section presents a comprehensive reliability study on the SFCL scheme, including analysis of the network stability, current limiting performance, and recovery characteristics of the SFCL both with and without the inclusion of a bypass switch. “Identification of the optimal location” section then describes a novel method for determining optimal locations of multiple SFCLs in a large scale electrical grid. Finally, the main conclusions of this paper are summarized in “Conclusion” section.

## SFCLs and topology of the power system

**Fig. 1:**
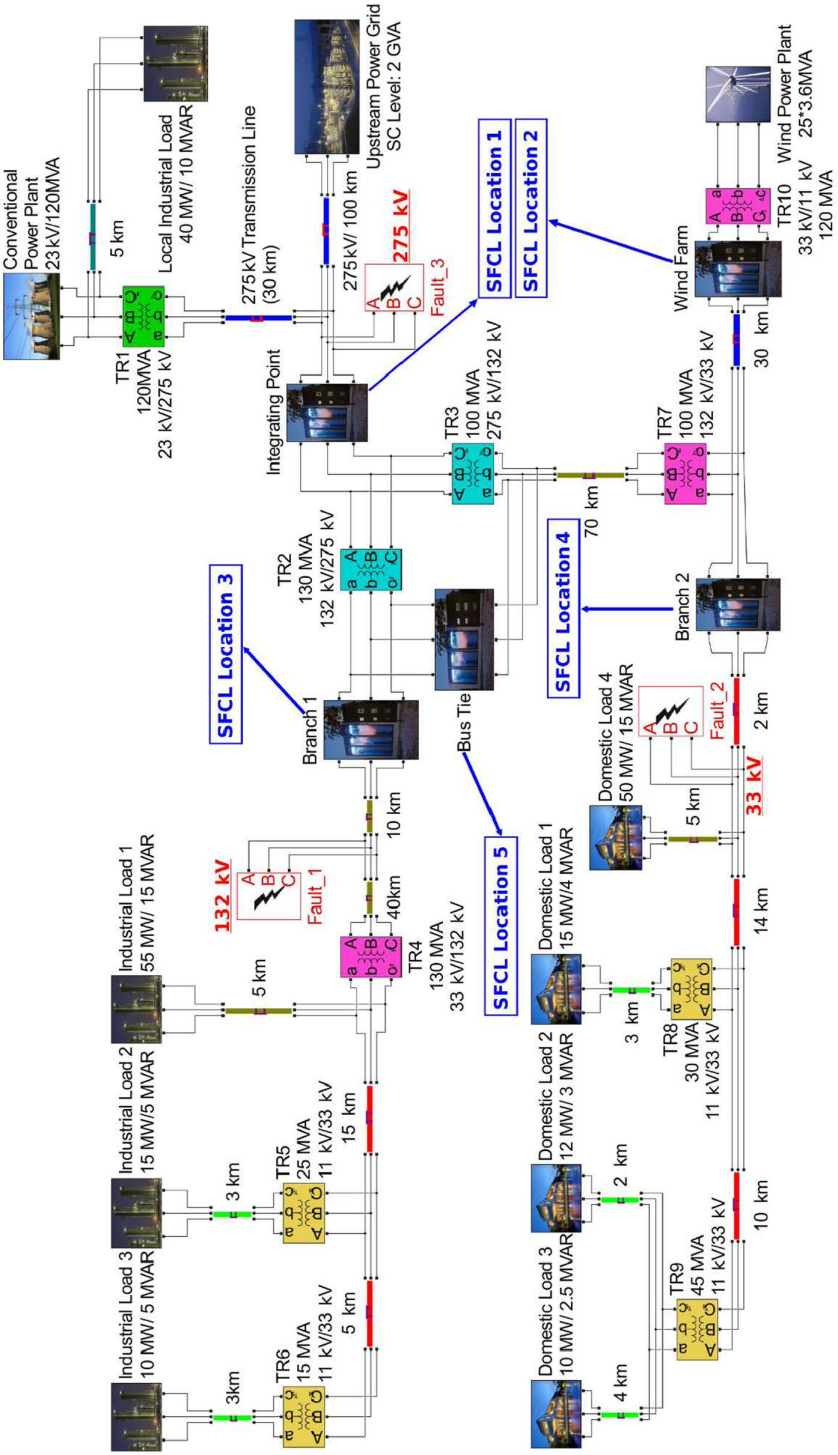
Power system model based on the UK grid standard as described in section “SFCLs and topology of the power system”. Three prospective fault positions and five prospective SFCL locations are illustrated

The topology of the modelled power system depicted in Fig. 1 was built based on the UK network standards (Butler 2001). The power system has a 120 MVA conventional power plant emulated by a three-phase synchronous machine, which is additionally connected to a local industrial load of 40 MW located 5 km away from the main power plant. Afterwards, the voltage level is boosted from 23 to 275 kV by a step-up transformer (TR1), from which the conventional power plant is connected to an upstream power grid rated with a short circuit level of 2 GW through a 130 km distributed-parameters transmission line. Then, the 275 kV high-voltage transmission system is split into two distribution networks. First, after the voltage level being stepped down to 33 kV by substations TR2 and TR4, the upper branch (industrial branch) supplies power to three industrial loads with a rated power of 55, 15, and 10 MW, separately. Likewise, the lower branch (domestic branch) is also connected to two step-down substations TR3 and TR7, with 70 km distance between them. The role of these two substations is reduce the voltage of the lower sub-grid to 33 kV, as it is the same voltage level rated by the interconnected 90 MVA wind power plant, which emulates the Rhyl Flats offshore wind farm located in North Wales, after being boosted by TR10. This offshore wind power plant is composed of twenty-five fixed-speed induction-type wind turbines each having a rating of 3.6 MVA, and is located 30 km away from its connecting point with the lower distribution network (Feng et al. 2010). After integration, the lower branch and the wind farm together provide electric energy to four domestic loads with a rated power of 50, 15, 12 and 10 MW, separately. Finally, the industrial branch and the domestic branch are connected through a bus-bar coupler, and the power system is balanced in a way that the current flowing through the bus-tie is only a few amperes during normal operation.

It is generally accepted that a three-phase (symmetric) short-circuit fault provokes the highest fault current among all possible faults, since it will cause the most drastic decrease of the system impedance. In order to ensure safe operation, the maximum current and electrodynamic withstand capabilities of electrical equipment are primarily designed according to this situation. Therefore, it is essential to simulate the behaviour of the power system under three-phase short-circuit fault. The symmetric faults were initialised at three potential locations marked as Fault 1 (132 kV), Fault 2 (33 kV) and Fault 3 (275 kV), which represent prospective faults occurring at the industrial branch, the domestic branch, and the transmission system, respectively (see Fig. 1). Five positions for the installation of SFCLs were proposed as shown in Fig. 1, namely at: (1) the integrating point between the conventional power plant and the upstream power grid (Location 1), (2) the interconnection between the wind farm and the port of domestic branch (Location 2), (3) the industrial loads branch (Location 3), (4) the domestic loads branch (Location 4), and (5) the bus-tie coupling the two distribution networks (Location 5).

Identical single phase SFCLs were implemented for each one of the three phases of the system, as each phase of the SFCL is only triggered by the current flowing through its own phase. However, under symmetric faults, each phase of the SFCL will quench slightly asynchronously within the first cycle of the fault current, leading to an instantaneous imbalance between the phases (Blair et al. 2012). Hence, for all types of faults and at diverse locations, independent modules for each one of the three phases have been considered, in order to allow for an accurate simulation of the effects of an SFCL on the overall power grid. Two different models were considered to emulate the SFCL performance, as described below.

### Step resistance SFCL

**Fig. 2:**
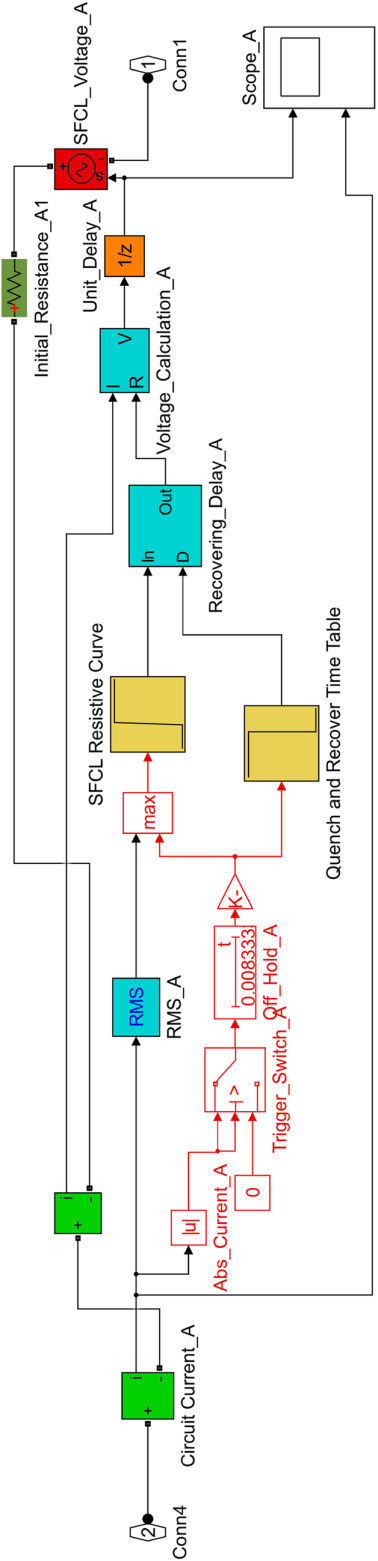
One phase of the step resistance SFCL model

The current limiting performance of the developed step resistance SFCL model is dominated by five predefined parameters: (1) triggering current; (2) quenching resistance; (3) quenching time, which has been assumed to be equal to 1 ms in accordance with Refs. Sung et al. (2009) and Alaraifi et al. (2013; 4) a normal operating resistance of 0.01 $\Omega$; and (5) a recovery time of 1 s. The values of the triggering current and quenching resistance are not provided in this section since they vary with the location of the SFCL. The structure of the step resistance model is illustrated in Fig. 2.

The operating principle of this model can be summarised as follows: first, the SFCL model calculates both the absolute and the RMS values of the flowing current. If both values are lower than the triggering current, the model will consider the SFCL in the superconducting state and insert a normal operating resistance (0.01 $\Omega$) into the grid. Otherwise, if either the absolute value or the RMS value of a passing current exceeds the triggering current level, the output resistance will be increased to the quenching resistance after the predefined quenching time. Lastly, if the current flowing through the SFCL model falls below the triggering current due to the clearance of the fault, the SFCL will restore its superconducting state after the recovery time.

### SFCL with E–J–T power law

The sudden change in the SFCL resistance can be macroscopically simplified into the *E*–*J* power law (Rhyner 1993), which can be divided into three sub-regions: the superconducting state defined by $E(T,t)<E_{0}$ and $T(t)<T_{c}$, the flux flow state defined by $E(T,t)>E_{0}$ and $T(t)<T_{c}$, and the normal conducting state defined by $T(t)>T_{c}$, with $T_{c}$ the critical temperature of the Bi2212 bar, and $E_{0}=1\times 10^{-6}\hbox {V}\cdot \hbox {m}^{-1}$ (Langston et al. 2005; Blair et al. 2012; Bock et al. 2015). All three sub-regions follow different power laws, the combination of which forms the *E*–*J* characteristics of the SFCL as follows:

1 $$\begin{aligned} E(T,t)= {\left\{ \begin{array}{ll} E_{c}\left( \dfrac{J(t)}{J_{c}(T(t))}\right) ^{n} , &\quad \text {for } E(T,t)<E_{0} \text { and } T(t)<T_{c},\\ E_{0}\left( \dfrac{E_{c}}{E_{0}}\right) ^{m/n}\left( \dfrac{J_{c}(77 K)}{J_{c}(T(t))}\right) \left( \dfrac{J(t)}{J_{c}(77 K)}\right) ^{m} , &\quad \text {for } E(T,t)>E_{0} \text { and } T(t)<T_{c},\\ \rho (T_{c})\dfrac{T(t)}{T_{c}}J(t) , & \quad\text {for } T(t)>T_{c}, \end{array}\right. } \end{aligned}$$

where,

2 $$\begin{aligned} J_{c}(T(t))=J_{c}(77K)\dfrac{T_{c}-T(t)}{T_{c}-77},\quad {\text{for}} \quad J>J_{c}. \end{aligned}$$

When modelling the SC state, we used $n=9$ in accordance with Refs. Buhl et al. (1997), Paul and Meier (1993), Herrmann et al. (1996), Bock et al. (2005), Noe et al. (2001) and $m=3$ for the flux flow state in good agreement with the experimental data reported in Refs. Paul et al. (2001) and Chen et al. (2002). In addition, we have assumed that the normal conducting state resistivity is a linear function of temperature when $T(t)> T_{c}$, with $\rho (T_{c}) = 7\times 10^{-6}\,\Omega$ for Bi2212 bars (Elschner et al. 2001). Furthermore, the relationship between the critical current density and the temperature was also set to be linear, as in Eq. (2), as this has been proven by Kozak et al. for the specific case of Bi2212 compounds (Kozak et al. 2005). To complete the SFCL model, a CuNi alloy $(\rho =40\,\mu \Omega \cdot m)$ resistor was connected in parallel with the superconductor on the basis of the project disclosed in Ref. Rettelbach and Schmitz (2003). This shunt resistance can protect the superconducting material from being damaged by hot spots that develop under limiting conditions, and furthermore prevents over-voltages that may possibly appear if the quench occurs too rapidly (Noe and Steurer 2007; Bock et al. 2004). Finally, by assuming that the SC composite is homogeneous, the thermal modelling of the SFCL considers the first order approximation of the heat transfer between the superconductor and the liquid nitrogen bath is as follows:

3 $$\begin{aligned} R_{SC}&= \dfrac{1}{2\kappa \pi d_{SC}l_{SC}} , \end{aligned}$$

4 $$\begin{aligned} C_{SC}&= \dfrac{\pi d_{SC}^{2}}{4}l_{SC}c_{v} ,\end{aligned}$$

5 $$\begin{aligned} Q_{generation}(t)&= I(t)^{2}\times R_{SFCL}(t), \end{aligned}$$

6 $$\begin{aligned} Q_{cooling}(t)&= \dfrac{T(t)-77}{R_{SC}}, \end{aligned}$$

where $R_{SC}$ stands for the thermal resistance from the SC material to its surrounding coolant, $C_{SC}$ is the specific heat of Bi2212  (Meerovich and Sokolovsky 2007), $c_{v}=0.7\times 10^{-6} J/(m^{3}\cdot K)$, and

7 $$\begin{aligned} T(t)=77+\dfrac{1}{C_{SC}}\int _{0}^{t}[Q_{generated}(t)-Q_{cooling}(t)]dt. \end{aligned}$$

The SC is modelled as a cylindrical wire of length $l_{SC}$, which is adjusted at each installing location in order to limit the prospective fault current to the desired level. Likewise, the diameter $d_{SC}$ is regulated to ensure that the SFCL not only remains into the superconducting state during normal operation, but also quenches within a few milliseconds once a short-circuit fault occurs at some location on the grid. In practice, although the wire diameter cannot be modified after fabrication, one can connect several wires in parallel to achieve the expected current limiting performance (Blair et al. 2011), which allows us to use the previous approaches.

## Network stability, current limiting performance, and recovery characteristics

**Fig. 3:**
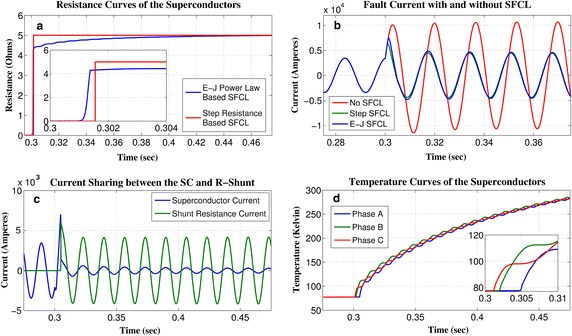
Performance comparison between the step SFCL model and the *E*–*J* power law based SFCL model: **a** resistance growth, **b** fault current characteristics, **c** current distribution in the SFCL, **d** temperature curves of each phase. The displayed insets in subplots **a** and **d** are measured in the corresponding units of the main plot

 In order to compare the fault current limitation properties of the two SFCL models, in Fig. 3 we present the results for a three-phase to ground fault with negligible fault resistance when it is initialised at the domestic network (Fault 2), and a single SFCL is installed next to the fault position (Location 4). Figure 3a illustrates that the step resistance model and the *E*–*J* power law based model both respond almost simultaneously to the occurrence of a short-circuit fault. However, as the SFCL needs ~2 ms to fully quench due to its *E*–*J* characteristic and dynamic temperature (Fig. 3d), the first peak reduction gained onto the step resistance model is overestimated by 11% (7.6 and 6.5 kA for the two SFCL models, respectively. 10 kA without SFCL), as shown in Fig. 3b. In addition, the shunt resistor diverts the major portion of the fault current after the superconductor develops its normal state (Fig. 3c). Therefore, the shunt resistance effectively lowers the thermal stress on the HTS wire, simultaneously preventing damages by overheating, whilst the recovery time is reduced (Morandi 2013).

**Fig. 4:**
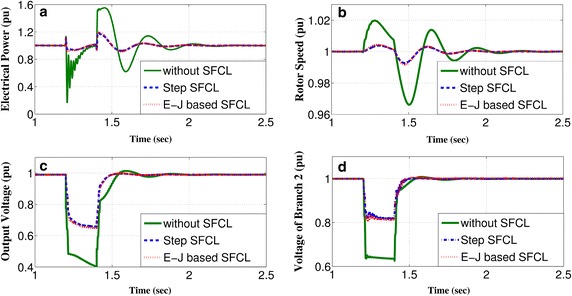
Generator parameters and voltages of branch 2 in response to a 200 ms three-phase to ground fault at branch 1

Initial tests without integration of the SFCL model have confirmed that the power system operates at the rated state during normal operation. Then, under occurrence of three-phase to ground faults at Fault-1, Fault-2 and Fault-3 (see Fig. 1), the short-circuit currents were measured at the integrating point (Location 1), wind farm (Location 2), branch 1 (Location 3) and branch 2 (Location 4), such that the instantaneous fault current can be described by:

8 $$\begin{aligned} i_{k}&=\underbrace{I_{pm}sin(\omega t+\alpha -\beta _{kl})}_{\text {periodic component}} \nonumber \\ &\quad+ \underbrace{[I_{m}sin(\alpha -\beta )]-I_{pm}sin(\alpha -\beta _{kl})]e^{-\dfrac{t}{\tau _{k}}}}_{\text {aperiodic component}}, \end{aligned}$$

where $I_{m}$ is the amplitude of the rated current of the power grid, $\phi$ and $\phi _{kl}$ represent the impedance angles before and after the fault, respectively, $\alpha$ defines the fault inception angle, $I_{pm}$ states the magnitude of the periodic component of the short-circuit current, and $\tau _{k}$ stands for the time constant of the circuit. Hence, the fault currents achieve their maximum values when $\alpha -\beta _{kl}=\pi (n+1) / 2$ with $n\in \mathbb {Z}$. This condition was implemented all through our study in order to consider the most hazardous fault scenarios, and the impact of the SFCLs on the generation side and the voltage stability of the grid. For instance, the response of the output electrical power, rotor speed, and terminal voltage for the conventional power plant (23 kV/120 MVA), and the voltage output at the domestic branch (Branch 2) for a scenario in which when a 200 ms three-phase to ground fault is applied at the industrial branch (Fault-1), after 1.2 s within normal operating conditions, as shown in Fig. 4.

Initially we have to consider the power system operation without the insertion of SFCLs. Under this scenario, the output electrical power drops sharply to 0.15 *pu* just after the fault incident (Fig. 4a), whilst the governors of the power plant, such as steam and hydro, still contribute with the same mechanical power to the rotors. Thus, a rapid acceleration of the rotors occurs due to this power imbalance, as shown in Fig. 4b. However, when an SFCL is installed at Branch 1 (Location 1), its high resistance state facilitates the SFCL to dissipate the excess generator power during the fault condition, hence improving the energy balance of the system and effectively reducing the variation of the rotor speed. Furthermore, considering the conventional equal-area criterion for stability issues (Sung et al. 2009; Kundur et al. 1994), the SFCL could improve the damping characteristics of generator speed and system frequency, as well as the system current, because the insertion of high resistance into the grid would significantly increase the damping ratio. Moreover, due to the short-circuit fault of Branch 1, a sharp voltage drop (Fig. 4c, d) can be seen at both the power plant terminal (0.5 *pu*) and the non-faulted Branch 2 (0.35 *pu*). Then, by introducing the SFCL, which acts as a voltage booster, the observed voltage dips are mitigated by 40 and 50%, respectively. This improvement allows the healthy parts of the system (without the fault inception) to be less affected, and makes integration of an SFCL a reliable fault ride-through scheme.

**Fig. 5:**
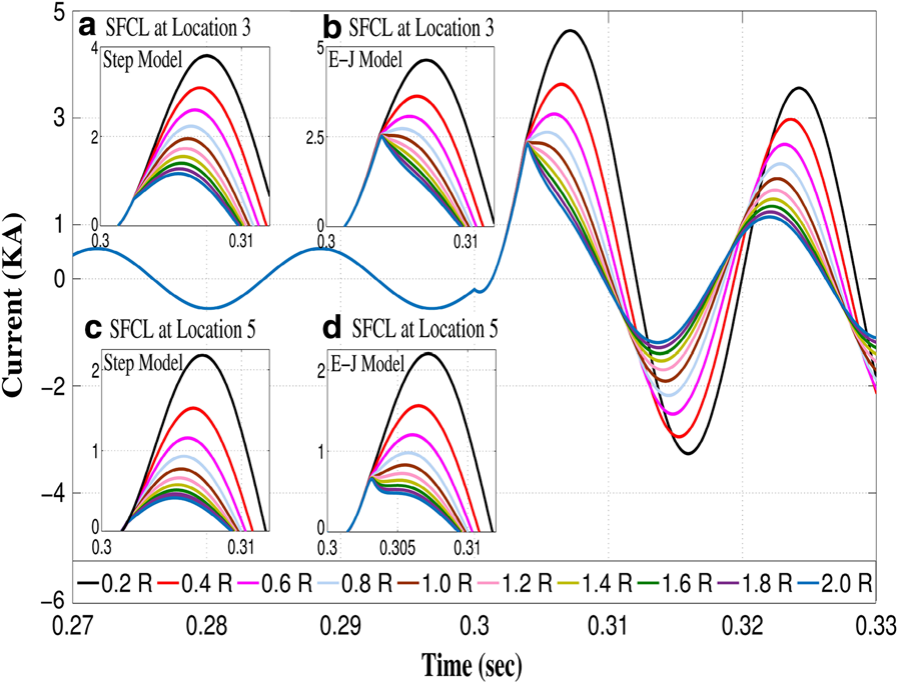
Current curves of phase A under a branch 1 fault (Fault 1) when the SFCL resistance increases from 0.2 R to 2.0 R

Without the protection of the SFCL, a 200 ms short-circuit fault was initiated in Branch 1 (Fault 1) in order to study the relationship between the current limiting performance of an SFCL and the maximum normal resistance. First, without the protection of the SFCL, simulation results have shown that the first peak of the current flowing into Branch 1 reached ~3.8 kA, which is ~6.8 times higher than the rated value (560 A). Then, after installation of the SFCL, a considerable reduction of the fault current was observed as shown in Fig. 5. The insets (a) and (b) on this figure illustrate the variation of the limited current when the two SFCL models (step resistance, and *E*–*J*–*T* power law) were integrated at Branch 1 (Location 3).

For the step-resistance model, with the SFCL resistance increasing from 0.2 R to 2 R $(R=30\,\Omega$) during the quenched state, the peak value of the fault current gradually decreased from ~3.8 to ~1.2 kA, showing a small displacement of the peak values. However, in the case of the *E*–*J*–*T* power law model, a noticeable *kink* appeared at 2.5 kA, when the maximum resistance of the SFCL was greater than 1 *R*. Remarkably, this distinctive *kink* can be interpreted as the threshold value for the maximum reduction of the fault current for an SFCL which cannot be determined with any other model, to the best of the knowledge of the authors. To illustrate the difference, the step-model resistance predicts a continuous decrease on the first peak of the fault current as *R* increases (Fig. 5a, c), contrary to what is observed with the more realistic *E*–*J*–*T* model (Fig. 5b, d), which predicts that no matter the increment of the SFCL resistance, after a certain value it can only limit the first peak of the fault current to a well defined threshold. For instance, for the case illustrated in Fig. 5, we have determined that on the instant that the *kink* appears, the current curves overlap at about 2.5 kA, defining hence, the maximum peak reduction of the fault current at this location (Location 3), and therefore an optimal SFCL resistance. It is worth mentioning that the characteristic kink is also observed when the SFCL is located at any other position, e.g., at the bus-tie (Fig. 5c, d), which validates the generality of our statement. Thus, in terms of economic considerations, it represents a very valuable result for distribution operators as it allows to state a maximum threshold on the required size for the capacity of the SFCL, minimising material investments for specific locations as beyond this threshold no further reduction of the first peak of the fault currents can be achieved.

**Fig. 6:**
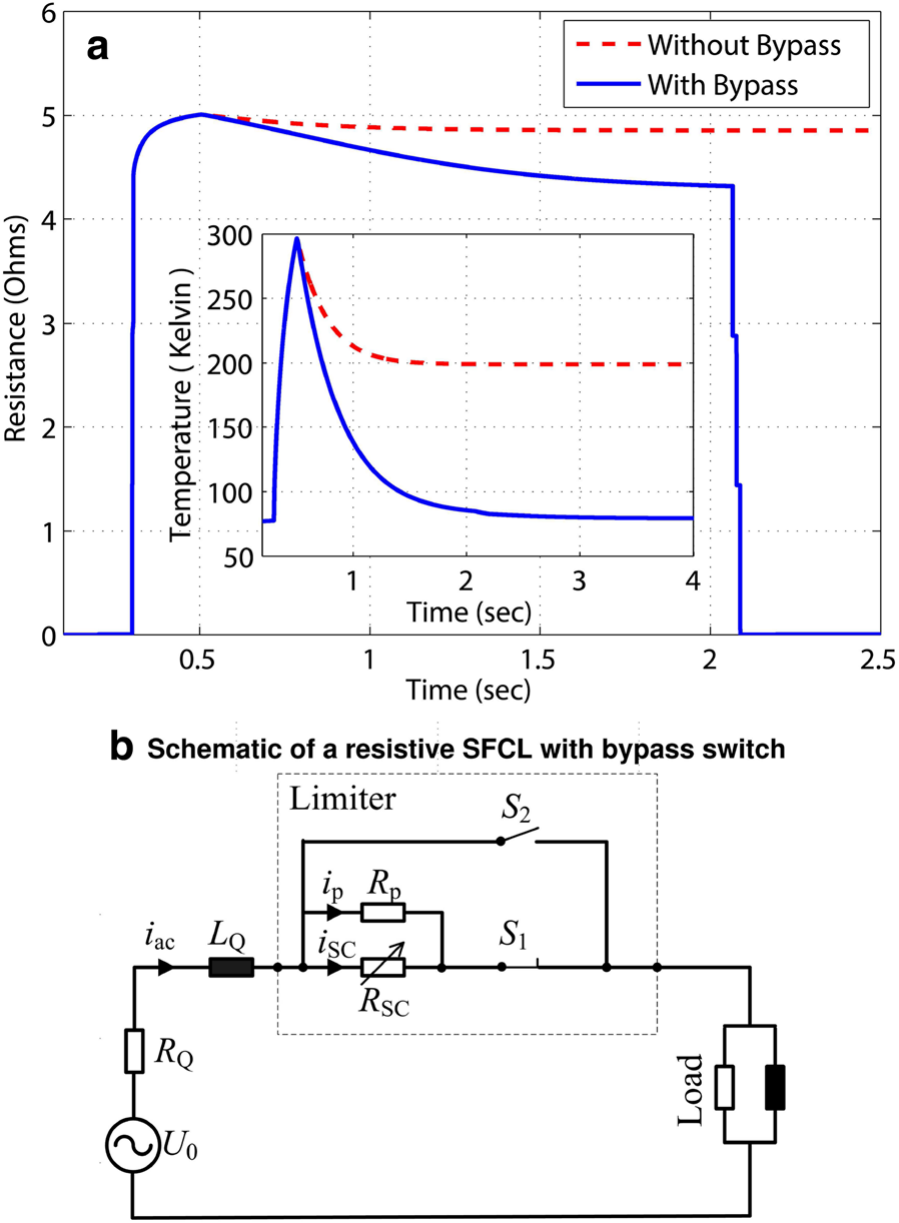
SFCL resistance and temperature dynamics with and without the assistance of the Bypass switch strategy shown in the *bottom* of the figure

Although the passive transition of the SC material and the high normal resistance enables the SFCL to limit the fault current before attaining its first peak, in some cases the recovery characteristics of the SFCL need to be improved because the SC may need several minutes to restore its superconducting state under load conditions. For instance, if a fault event quenches a single SFCL located at the domestic branch, it may take more than 300 seconds to recover once the fault current has been cleared. Therefore, in order to decrease the recovery time of the SFCL we have connected a bypass switch parallel to both the SC and the shunt resistance (Melhem 2011). Thus, when the SFCL can quickly recover the superconducting state under load conditions, the switch *S*1 remains closed after the fault is cleared. However, if the SFCL cannot be automatically recovered within a few seconds, then switch *S*2 can be closed and switch *S*1 instantaneously opens to quickly disconnect the SC from the system. This allows the SC to undergo its recovery process without further accumulation of heat, as shown in Fig. 6 for an SFCL installed at Location 2 after encountering a 0.2 s three-phase to ground fault at the domestic branch (Fault 2). For this case, and without applying the bypass switch strategy, a certain amount of current will continue passing through the SFCL after the clearance of the fault. This flow of current keeps continuous to generate heat inside of the superconductor, which significantly slows down the decrease of temperature, and hence delays the recovery of the SFCL by over five minutes. However, with a properly designed control scheme, the *E*–*J*–*T* model can open the switch *S*1 and close the switch *S*2 at the moment that the fault ends, thus transferring the current to the *S*2 branch. In fact, by using this method we have determined that the recovery time can be reduced to less than 1.6 s without affecting the normal operation of the power grid. After the SC is restored to its superconducting state, the switches *S*1 and *S*2 act again to prepare the SFCL for the next fault. However, as it is not possible to foresee the location of a fault event, the optimal location for the installation of one or more SFCLs has to be assessed, being this the purpose of the following section.

## Identification of the optimal location

In order to attain an accurate estimation of the optimal location for the installation of one or more SFCLs, all possible SFCL combinations according to the five proposed locations depicted in Fig. 1, were analysed for the three different fault points. This resulted in a total of 31 allocation strategies, including five different schemes for the integration of a single SFCL (Locations 1–5), 10 dual combinations of SFCLs, 10 further combinations of three SFCLs, five combinations of four SFCLs, and finally the cooperation between all five SFCLs.

**Fig. 7:**
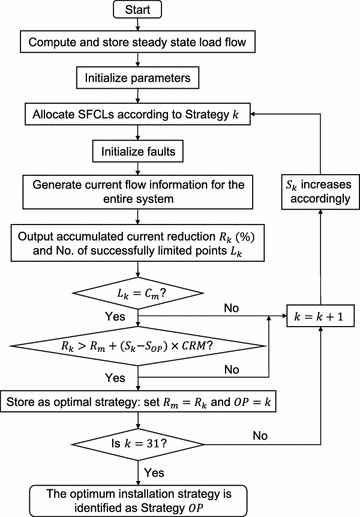
Flowchart of the algorithm for determining optimal installation strategy of SFCLs. Parameters being initialized during the third step: number of $k=1$; number of installed SFCL $S_{k}=1$; maximal current reduction $R_{m}=0$; current reduction margin of one additional SFCL$=CRM$; number of measured points $C_{m}$; optimal strategy $OP=0$

The current signals at both the wind farm terminal (Location 2) and the integrating point of the conventional power plant and the upstream power grid (Location 1) were measured for all three fault conditions (Fig. 1). We also analysed the current injection of the industrial branch (Location 3) and the domestic branch (Location 4) when faults happen at the two networks: Fault 1 and Fault 2, respectively. For the sake of brevity, we do not present the results for the measured current at the industrial branch when Fault 2 or Fault 3 occurs, because based on the analysis of the system impedance change, the magnitude of the current flowing into the industrial branch is actually reduced by the two faults to levels lower than the normal current, i.e., at this point the SFCL does not need to be triggered to protect this branch. The same argument applies to the domestic branch under Fault 1 and Fault 3 conditions. Our results are presented below in terms of the single or multiple SFCL strategies. The optimal SFCL installation scheme was found by following the algorithm shown in Fig. 7.

### Single SFCL installation

**Fig. 8:**
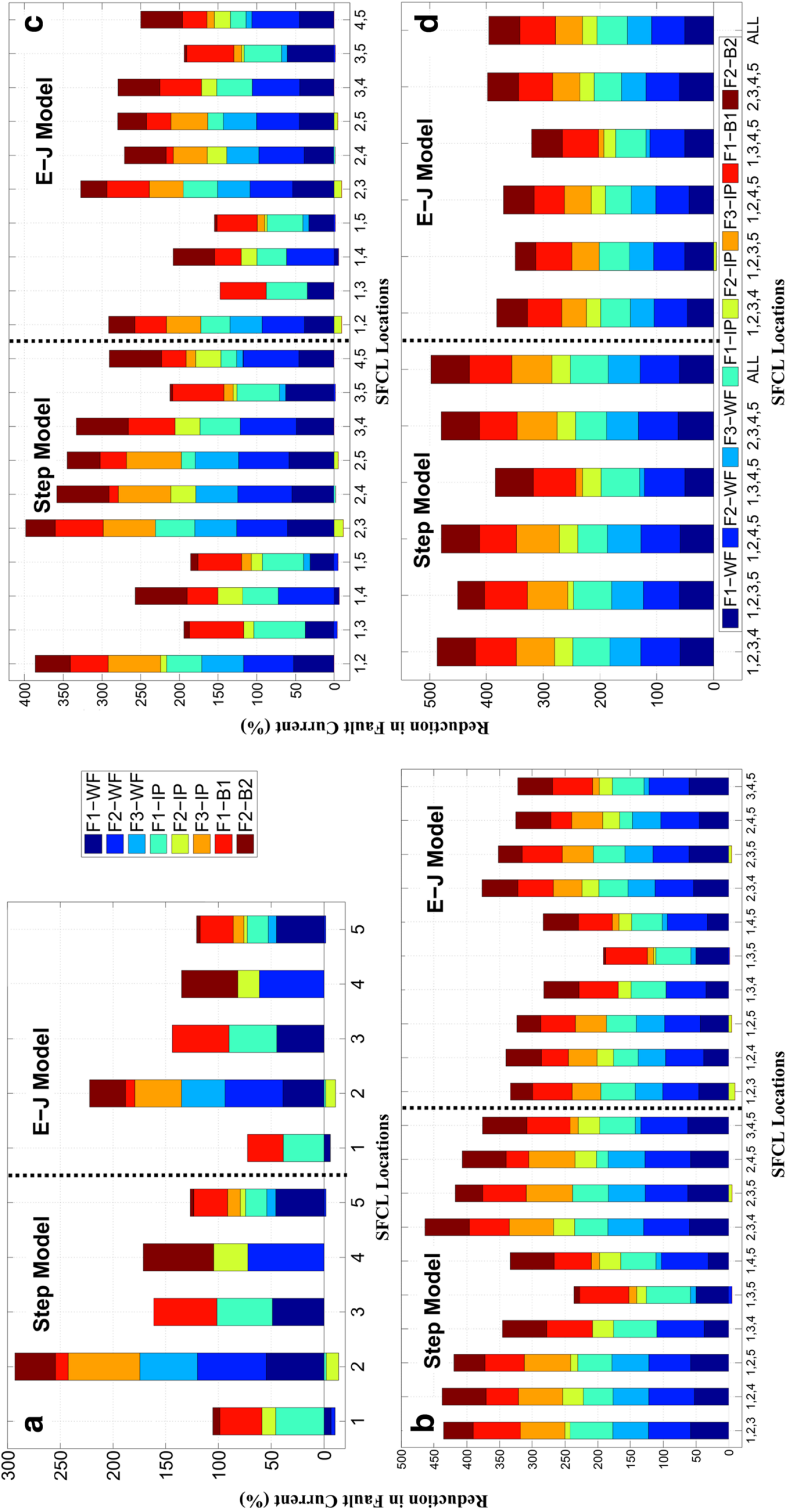
Reduction of the first peak of the fault current events at different locations, measured after the installation of a **a** single SFCL, **b** two SFCLs, **c** three SFCLs, and **d** up to four and five SFCLs installed at different locations

Figure 8a shows the reduction in the fault current under the three fault conditions illustrated in Fig. 1 when a single SFCL is installed at at the referred locations (Locations 1 to 5). For the sake of comparison, the size of the superconductor which has to be defined into the *E*–*J*–*T* power law model, was systematically adjusted so that it defined the same maximum resistance as the one used with the step resistance model. Thus, when the step resistance model was considered, the maximum reduction of the fault current was overestimated in comparison with the more realistic *E*–*J*–*T* model. For all five SFCL locations, the first peak of the fault current was always found to be lower in the first case. The reason for this difference is that, once the current exceeds the critical value of the SC, the SFCL described by the step resistance model directly jumps to the maximum resistance after the pre-defined response time, whilst in the *E*–*J*–*T* model the dynamic increase of the resistance depends not only on the passing current, but also on the temperature of the superconductor. Therefore, under the *E*–*J*–*T* model the SFCL cannot gain its maximum rated resistance before the first fault peak is reached, which leads to a relatively lower reduction of the fault current (~20%).

Based on both the SFCL models tested, the simulations performed generally showed a negative impact on the reduction of the fault peak at certain integration points when the SFCL was installed at Location 1 or Location 2. In these cases the fault current was actually increased by the insertion of a SFCL. In more detail, when the SFCL was installed beside the wind farm (Location 2), the sudden increase in the fault current flowing through the integrating point under Fault 2 (at the domestic branch) was caused by the abrupt change of the impedance of the power system. This SFCL entered the normal state, reducing the current output of the wind farm due to its rapid rise in resistance and hence, the conventional power plant and the upstream power grid were forced to supply a higher current to the faulted branch. Similar behaviour was obtained under the fault conditions F1 and F2 when the SFCL was installed at Location 2, and the current was measured at the integrating point (see Figs. 1, 8a). Furthermore, when a single SFCL was installed at Location 1 (integrating point), following the *E*–*J*–*T* model the SFCL can only limit the fault current in two cases, whilst with the simplified step-resistance the benefits of the SFCL can be overrated as it leads to a positive balance in up to four different fault conditions. This highlights the importance of finding a suitable optimal allocation strategy for the SFCLs under a wide number of fault conditions, and the need for considering adequate physical properties for the electro-thermal dynamics of the SC materials. It ultimately tries to fill the gap between acquired scientific knowledge and the demand for more reliable information from the standpoint of the power distribution companies. Thus, the final decision for an optimal location has to be made under the circumstance of having a twofold conclusion.

Firstly, the decision can be made according to the highest total reduction on the fault current passing through different points and under different fault circumstances as shown in Fig. 8a. There, it can be observed that for the eight most important cases combining the occurrence of a fault at certain positions and the measuring point for the current reduction, the SFCL installed at the port of the wind farm (Location 2) appears to be the best option, as in this case the fault current can be reduced in six of the eight different scenarios with an accumulated reduction of 290% from the step resistance model, and 220% from the *E*–*J*–*T* power law model, respectively. Nevertheless, this strategy has also an adverse impact on the remaining two other scenarios (F1-IP & F2-IP). Secondly, a decision can be made in terms of the overall performance for achieving positive impacts under the scope of any of prospective circumstances. In this sense, we have determined that placing the SFCL at Location 5, at the bus-tie between the industrial and domestic branches, is the most reliable option. An SFCL installed at the bus-tie is capable of reducing the harmonics and voltage dips, doubling the short-circuit power, and ensuring even loading of parallel transformers (Colmenar-Santos et al. 2016). Moreover, the recovery characteristics of the SFCL can also see benefit from this arrangement as after a quench of the SFCL, the bus-tie can be switched open for a short time (few seconds) to help the SFCL restore the superconducting state. However, a drawback of this switching strategy is that this measure may temporarily reduce the quality of the power supply, but a strong impact on the normal operation of the power system is not foreseen.

### Multiple installation of SFCLs

Firstly, a double protection strategy, the installation of two SFCLs in different grid positions, was assessed. According to both the step resistance model and the *E*–*J* power law based model, the highest fault current reduction was always achieved when the SFCLs were installed at Location 2 (wind farm) and Location 3 (industrial branch) simultaneously, accomplishing a 400 and 330% total fault limitation, respectively (Fig. 8b). Indeed, this arrangement can be considered a much better strategy in comparison to the results obtained when just a single SFCL was considered, as the total current limitation is improved by ~110%. Furthermore, contrary to the previous case, the current flowing through the integrating point when the fault occurs at the industrial branch (Fault 2) significantly decreased rather than having an adverse effect on the power system. Moreover, under this dual strategy the measured current reduction showed a balanced performance on all the different analysed cases, unlike the results obtained for when a sole SFCL was installed. In addition, if system operators measure the optimal strategy for the installation of two SFCLs in terms of the number of limited cases, different conclusions can be obtained under the framework of different physical models, e.g., when the step-resistance or the *E*–*J*–*T* power law model is considered. According to the step resistance model, installing the two SFCLs at either Locations 1 & 2 or Locations 4 & 5 produced a positive response to all eight measured fault conditions. When the SFCLs were installed at Locations 1 & 2, a better performance was obtained as the total reduction in the fault current (330%) was 40% greater than the performance obtained by SFCLs installed at Locations 4 and 5 (290%). However, when the *E*–*J*–*T* model was used, installing the SFCLs at Locations 1 & 2 increased the magnitude of the current at the integrating point under the occurrence of a fault in the domestic branch (Fault 2). This was due to the unsuccessful triggering of the SFCL at Location 1, as explained in the previous subsection. Therefore, from the point of view of the system operators, Locations 4 & 5 can be considered as the most reliable solution as it is the only combination capable of limiting all fault conditions and for all the considered scenarios.

Secondly, we added an additional SFCL to the grid to assess the overall performance of this new system. Figure 8c shows that most of the installation strategies for three SFCLs produced a reduction of the fault current in all eight measured scenarios. Both SFCL models agreed with the conclusion that the greatest reduction in the fault current was achieved when the SFCLs were installed simultaneously at the Locations 2, 3 and 4. This strategy showed a 470% total reduction using the step resistance model, and 375% using the *E*–*J*–*T* model, attaining a significant increase on the overall performance of the system by about 70 and 45%, respectively, in comparison with the best achieved performance when the dual SFCLs strategy was considered. Besides this huge improvement, the three SFCLs strategy could also respond positively to any fault conditions, which means installing three SFCLs can be considered the most reliable strategy for both overall fault current reduction and the number of cases exhibiting fault current reduction. Moreover, it was found that, under all fault conditions, the fault current levels of all measured points can be reduced to lower than the safety thresholds, which was set as three times of the normal current according to common practice. Until a significant reduction of the overall price of a SFCL is achieved, distribution network operators may not consider this strategy to be be cost-effective in terms of the initial investment, but given the expected reduction on the price of the second generation of high temperature superconducting wires, this decision can be seen as the most profitable strategy in terms of grid safety and reliability. However, a limit for the maximum number of the SFCLs required must also be established in order to guarantee the maximum benefits at minimum cost.

Thus, in Fig. 8c we show the performance comparison among five different scenarios when four SFCLs were installed into the power system. With four SFCLs working together, all of the combinations effectively limited the fault current for all eight studied cases, except for when the SFCLs were described using the *E*–*J*–*T* model and installed at Locations 1, 2, 3, and 5. Under this scheme the measured fault current increased when the fault was initialised at the domestic branch (Fault 2), due to the lack of action from the SFCL installed at Location 1. When the step resistance model was considered, the accumulated maximum reduction on the fault current was again overestimated, achieving a 480% reduction when the SFCLs were installed at Locations 1, 2, 3 and 4 or at 2, 3, 4 and 5. In comparison, Locations 2, 3, 4 and 5 produced a prospective reduction of 395% when the more realistic *E*–*J*–*T* model was considered. The maximum accumulated reduction of the fault current achieved by any of the strategies using four SFCLs was just over 10% more than the most effective of the strategies using three SFCLs. This enables us to define an upper limit for the number of SFCLs needed.

In order to verify our previous statement, we also studied the result of considering even one more SFCL, as there are five prospective locations for SFCL installations in the power grid displayed in Fig. 1. Compared to the last analysed case (4 SFCLs), the accumulated maximum reduction of the fault current reached a 15% greater reduction when the SFCLs were simulated using the step resistance model, but surprisingly no further improvement was obtained when the more realistic *E*–*J*–*T* model was incorporated. This important result can be understood as a consequence of the mutual influence between the integrated SFCLs, i.e., when the fault current passing through one SFCL is substantially decreased by the influence of the others, the rate of heat accumulation reduces accordingly, slowing down the rate of the temperature rise and hence reducing the resistance that the SFCL can develop before reaching the first peak of the fault.

**Table 1:** Optimal installation strategies for SFCLs according to the step-resistance and E–J power law models

*Step-resistance model*					
Maximum fault current reduction (%):	290	400	470	480	495
No. of measuring conditions with/without FCR:	6/2	7/1	8/0	8/0	8/0
Number of installed SFCLs:	1	2	3	4	5
SFCLs’s locations:	2	2, 3	2, 3, 4	1, 2, 3, 4$^{\mathrm{a}}$	1, 2, 3, 4, 5
No. of measuring conditions with/without FCR:	7/1	8/0	8/0	8/0	8/0
Accumulated FCR (%) for max. no. of measuring conditions:	130	330	470	480	495
Number of installed SFCLs:	1	2	3	4	5
SFCLs’s locations:	5	1, 2	2, 3, 4	1, 2, 3, 4	1, 2, 3, 4, 5
*E–J power law model*					
Maximum fault current reduction (%):	220	330	375	395	395
No. of measuring conditions with/without FCR:	6/2	7/1	8/0	8/0	8/0
Number of installed SFCLs:	1	2	3	4	5
SFCLs’s locations:	2	2, 3	2, 3, 4	2, 3, 4, 5	1, 2, 3, 4, 5
No. of measuring conditions with/without FCR:	7/1	8/0	8/0	8/0	8/0
Accumulated FCR (%) for Max. No. of measuring conditions:	120	250	375	395	395
Number of installed SFCLs:	1	2	3	4	5
SFCLs’s locations:	5	4, 5	2, 3, 4	2, 3, 4, 5	1, 2, 3, 4, 5

The maximum fault current reduction (FCR) value (per case) has been calculated as the sum of the percentage reductions of the fault current measured at the wind farm output, the integrated point, and branches 1 and 2, for the three fault conditions shown in Fig. 1. The fault current was not reduced at all measuring locations, as shown in Fig. 8. Therefore, the table also shows the values for the accumulated fault current reduction when the fault current was reduced for the greatest number of measuring conditions

^a^ Same performance is achieved when the four SFCLs are located at the positions 2, 3,4, and 5

Table 1 summarizes the optimal allocation strategies and the corresponding performances of the SFCLs modelled during our study. The preferable locations for the installation of the SFCLs have been determined in terms of the two identified standards: (1) the maximum accumulated fault current reduction, and (2) the maximum number of measuring conditions that could be limited. The results in the table are categorised by the number of SFCLs required by each strategy, and also the physical models used to emulate the characteristics of the SFCLs. In all the cases the step resistance model led to an overestimation of the actual performance figures achievable by the SFCLs when more realistic physical properties were considered. Finally, when the strategy is to maximize the benefits from installing only one or two SFCLs, a compromise must be made between increasing the fault current reduction, and maximizing the actual number of measuring conditions where the fault current can be limited. Therefore, based upon the comprehensive study presented in this paper, we conclude that the optimal installation strategy is the installation of a maximum of three SFCLs at Locations 2, 3, and 4, as this strategy produced the maximum reduction of the fault current for all fault conditions, and the addition of further SFCLs did not represent a significant enough improvement to justify the increased cost.

## Conclusion

The superconducting fault current limiter is a promising device that can limit the escalating fault levels caused by the expansion of power grids and the integration of renewable energy sources. This paper presents a comprehensive study on the performance and optimal allocation analysis of resistive type SFCLs inside of a power system based on UK network standards. In order to assess the impact of incorporating SC material properties on the performance of SFCLs, two different models were used throughout the study. First, the active operation of an SFCL was modelled using a Heaviside function. Second, a more realistic model was used to simulate the operation of an SFCL, taking into consideration the proper E–J characteristics of the superconducting material and dynamic temperature evolution. Independently of the model used, we have proven that SFCLs can effectively improve the damping characteristics of the generation system, and can mitigate voltage dips at the grid. However, we have shown that although computing time can be reduced when step-resistance models are used, such simplifications lead to strong overestimations of the actual prospective performance of the SFCL, in terms of the maximum reduction on the fault current and its correlated normal resistance. Thus, this comparison led us to the conclusion that adequate physical properties for the electro-thermal dynamics of the SC materials has to be considered in order to accurately predict behaviour of SFCLs inside a power system.

A systematic study was then performed using the prospective strategies for the installation of one or more SFCLs. We have proven that installing more SFCLs does not necessarily mean better overall performance. For our power system model, the simultaneous use of three SFCLs that installed at Locations 2, 3, and 4, is the best protection strategy in terms of the performance, economic efficiency and the reliability of the overall grid. In order to draw this conclusion, all the potential combinations of two, three, four, and five SFCLs were studied under a wide number of fault scenarios and measuring strategies.

## Abbreviations

SFCLs: superconducting fault current limiters
DGs: distributed generations
TR: transformer
